# Multistate Outbreak of Human *Salmonella* Typhimurium Infections Linked to Contact with Pet Hedgehogs — United States, 2011–2013

**Published:** 2013-02-01

**Authors:** Nicola Marsden-Haug, Stephanie Meyer, Sally A. Bidol, Jennifer Schmitz, Wright Culpepper, Casey Barton Behravesh, Jamae Morris, Tara Creel Anderson

**Affiliations:** Communicable Disease Epidemiology, Washington State Dept of Health; Acute Disease Investigation and Control Section, Infectious Disease Epidemiology, Prevention, and Control Div, Minnesota Dept of Health; Michigan Dept of Community Health; Animal Care, Animal and Plant Health Inspection Svc, US Dept of Agriculture; Div of Foodborne, Waterborne, and Environmental Diseases, National Center for Emerging and Zoonotic Infectious Diseases; EIS officers, CDC

CDC is collaborating with the U.S. Department of Agriculture’s Animal and Plant Health Inspection Service (USDA-APHIS) and state health departments to investigate an outbreak of human *Salmonella* Typhimurium infections with an indistinguishable pulsed-field gel electrophoresis pattern linked to contact with pet hedgehogs. This outbreak strain is historically rare, with only one to two cases reported via PulseNet (the national molecular subtyping network for foodborne disease surveillance) annually since 2002. Since 2011, an increasing number of cases have been detected. PulseNet identified 14 human isolates in 2011, 18 in 2012, and two in 2013.

Since January 2012, a total of 20 persons infected with the outbreak strain of *Salmonella* Typhimurium have been reported from eight states: Alabama (one), Illinois (one), Indiana (one), Michigan (three), Minnesota (three), Ohio (three), Oregon (one), and Washington (seven). Illness onset dates ranged from December 26, 2011, to December 31, 2012. The median patient age was 13 years (range: <1–91 years); 55% of patients were female. Four patients were hospitalized. One death associated with *Salmonella* infection has been reported. Fourteen out of 15 patients (or their proxies) reported direct or indirect contact between the patient and a hedgehog during the week before illness onset. The hedgehogs were purchased from various hedgehog breeders, many of whom were USDA-APHIS licensed, in several states. CDC, USDA-APHIS, and state health departments currently are collaborating to conduct a traceback investigation of hedgehogs purchased from USDA-APHIS licensed breeders by members of the households of ill persons.

Salmonellosis is most commonly foodborne; however, contact with infected animals and their environments also can cause illness (1). Salmonellosis has been linked with pet hedgehogs previously (2,3). Children aged <5 years, elderly persons, and immunocompromised persons are at increased risk for severe illness. Infections can result from direct contact with hedgehogs during routine care and indirect transmission through contact with objects (e.g., cages, toys, or bedding) or household surfaces that come in contact with infected hedgehogs.

Hand washing with soap and water after handling hedgehogs, especially before handling food or drinks, can reduce the risk for infection. Any equipment or materials associated with hedgehog care (e.g., feed, water, and bathing containers) should be cleaned outside the home. Detailed safe handling instructions for hedgehogs should be provided at the point of sale, and owners should ensure that anyone in direct or indirect contact with hedgehogs is aware of proper precautions to prevent *Salmonella* transmission. Additional information is available at http://www.cdc.gov/salmonella/typhimurium-hedgehogs-09-12.
